# Resolving oligomeric states of photoactivatable proteins in living cells via photon counting histogram analysis

**DOI:** 10.1016/j.isci.2025.113848

**Published:** 2025-10-23

**Authors:** Tyler Camp, Zixiao Li, Yushan Li, Teak-Jung Oh, Kai Zhang

**Affiliations:** 1 Department of Biochemistry, University of Illinois Urbana-Champaign, Urbana, IL, USA; 2 STC-QCB center, University of Illinois Urbana-Champaign, Urbana, IL, USA; 3 Cancer Center at Illinois, University of Illinois Urbana-Champaign, Urbana, IL, USA; 4 Center for Biophysics and Quantitative Biology, University of Illinois Urbana-Champaign, Urbana, IL, USA; 5 Neuroscience Program, University of Illinois Urbana-Champaign, Urbana, IL, USA; 6 Beckman Institute for Advanced Science and Technology, University of Illinois Urbana-Champaign, Urbana, IL, USA

**Keywords:** molecular interaction, protein, structural biology

## Abstract

Oligomerization of photoactivatable proteins underlies many optogenetic strategies, yet their assembly states remain difficult to quantify in living cells. Here, we applied photon counting histogram analysis to directly measure the oligomerization of widely used optogenetic modules, *Vaucheria frigida* Aureochrome light-oxygen-voltage (VfAuLOV) and *Arabidopsisthaliana* cryptochrome 2 (AtCRY2), in living HEK293T cells. Oligomerization of both photoactivatable protein variants is concentration-dependent in cells. VfAuLOV primarily forms dimers, whereas AtCRY2 transitions into tetramers at concentrations above 1,000 nM, consistent with cryoEM structures. Human CRY2 exhibits light-independent oligomerization, while inactive AtCRY2 mutants (D387A and R439L) remain monomeric in light or darkness. Surprisingly, the constitutively active AtCRY2(W374) mutant still undergoes light-mediated oligomerization. The extent of light-induced lytic cell death correlates with the oligomerization state of these proteins when fused to receptor-interacting serine/threonine protein kinase 3. This study establishes a quantitative framework to resolve protein assembly dynamics in living cells, advancing mechanistic understanding of optogenetic tools and broadening their applications in cell signaling research.

## Highlights

• PCH analysis quantifies photoactivatable protein oligomerization states in living mammalian cells
• AuLOV and AtCRY2 oligomerization depends on protein expression levels
• Inactive AtCRY2 mutants remain monomeric regardless of light conditions
• The active AtCRY2(W374A) mutant retains blue light responsiveness

## Graphical abstract

**Figure:**
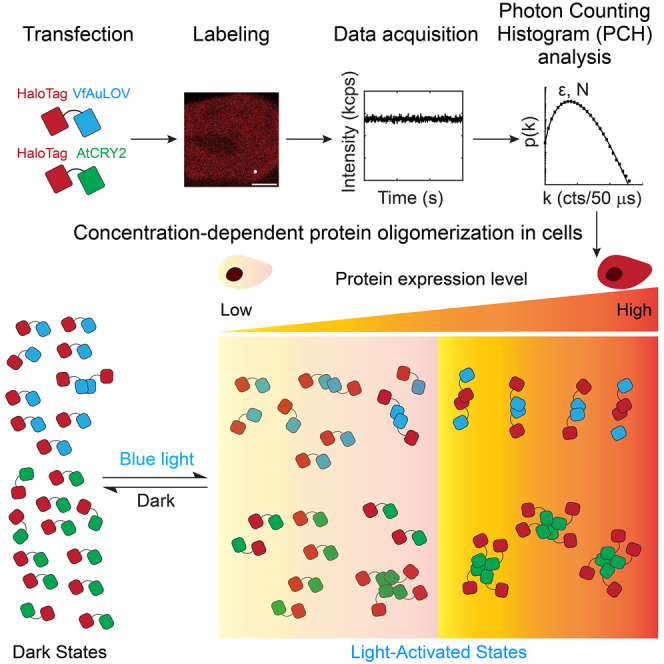

## Introduction

Biomolecular condensates play a crucial role in catalyzing chemical reactions in living cells. Besides well-established intrinsically disordered proteins that can initiate the assembly of the condensates,^1^ photoactivatable proteins were used to modulate phase transition through light-induced protein-protein interactions, an optogenetic approach.^2^ Dimerization and oligomerization are fundamental principles in optogenetic system design.^3^ While *in vitro* methods, such as biochemical assays, crystallography, and cryo-electron microscopy (cryo-EM), provided insights into the oligomerization state of photoactivable proteins, a quantitative understanding of these dynamics in living cells is currently lacking. Such insights prove critical for rational design and practical implementation of optogenetic strategies in multicellular organisms, motivating a reliable, quantitative approach to resolve the *in situ* protein oligomerization state.

Fluorescence fluctuation spectroscopy (FFS), which relies on temporal fluctuations in fluorescence intensity, recovers information on fluorophore concentration and average oligomer size in living cells. Techniques falling under the FFS umbrella, such as spatial intensity distribution analsysis (SpIDA)^4^ and fluorescence intensity fluctuation analysis,^5^ have been applied to study protein diffusion and oligomerization.^6^^,^^7^ FFS-based experiments are particularly useful for studying assemblies larger than dimers that are challenging to resolve by FRET.^8^ Photon counting histogram (PCH) analysis^9^ and related single-point techniques, wherein the fluorescence signal is measured at a single spot over time using focused, stationary laser excitation, have successfully resolved dimers,^10^ pentamers,^11^ and even much larger assemblies of more than several hundred protein subunits per complex,^12^ all within living cells. Here, we used PCH analysis to resolve the oligomerization state of photoactivatable proteins in living cells. We found that the HaloTag, a self-labeling protein tag, is a better probe for PCH analysis than conventionally used fluorescence proteins,^13^ due to improved brightness and photostability of chemically synthesized small-molecule fluorescent probes.

To gain insights into light-mediated dimerization/oligomerization of photoactivatable proteins, we selected two commonly used homotypic dimerization/oligomerization photoactivatable proteins: the light-oxygen-voltage (LOV) domain of *Vaucheria frigida* Aurochrome1 (herein VfAuLOV)^14^^,^^15^^,^^16^ and the photolyase homology region of cryptochrome 2 from *Arabidopsis thaliana* (herein AtCRY2).^17^ Additional derivatives of these proteins, including bZip-VfAuLOV,^18^ inactive AtCRY2 (D387A) and AtCRY2 (R439L) mutants, the constitutively active AtCRY2 (W374A) mutant, as well as the human CRY2 photolyase homology region (HsCRY2), were also measured. Previous structural studies used size-exclusion chromatography to resolve aggregation *in vitro*,^18^ together with the structures of AtCRY2 resolved by cryo-EM^19^^,^^20^^,^^21^ or X-ray crystallography.^22^^,^^23^ However, only low-resolution descriptions of photo-induced protein-membrane associations^24^^,^^25^^,^^26^ or homo-hetero-oligomerization^27^^,^^28^^,^^29^^,^^30^ were available in cells. Measuring the oligomerization state of these optogenetic systems in living cells would therefore fill an important gap and inform future cellular engineering efforts toward a mechanistic understanding of the relationship between protein concentration, oligomerization state, and signaling outcomes.

## Results

### Fluorescent proteins show distinct photostability in mammalian cells

**Figure 1:**
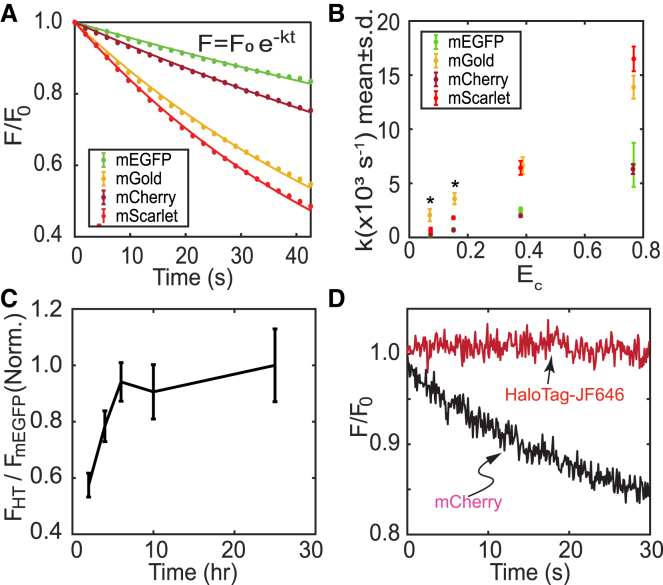
Benchmark fluorophore photophysics in living HEK293T cells (A) Representative photobleaching trajectories from cells expressing mEGFP, mGold, mCherry, and mScarlet after repeated scanning on a commercial laser scanning confocal microscope. (B) Photobleaching rate constants recovered from the bleaching of individual cells as a function of the relative excitation power. Asterisks mark the apparent rate constants for mGold excited at low power, where a single-exponential model did not fully describe the fluorescence decay. (C) Optimization of HaloTag-JF646 staining protocol. Cells expressing a fusion of HaloTag and mEGFP were stained with JF646 for the indicated times, destained, and imaged using a plate-based fluorescence imaging system (*N* = 4 cells). (D) Single-spot photobleaching trajectories from cells expressing HaloTag-JF646 (red) or mCherry (black) that were excited at a similar power. (A and D) Data are normalized to the intensity at t = 0 (*N* = 3 cells for each laser power and each fluorophore). For (B) and (C), data are represented as Mean ± SD. Also see Figure S1.

Conventional FFS often uses fluorescent proteins (FPs) as the fluorophore. An optimal FP should have high brightness (proportional to the product of absorption coefficient and quantum yield) and photostability. Thus, we first compared the photostability of various commonly used FPs, including mEGFP, mGold, mCherry, and mScarlet. HEK293T cells were transfected with each plasmid and incubated for 16 h before imaging. A small region was selected from each cell to analyze the photobleaching data, and the average intensity was measured for each frame. Representative photobleaching trajectories of each FP are shown in Figure 1A. The photobleaching rate constant for each FP was calculated by fitting each curve with a single-exponential decay. To calibrate the effect of distinct excitation wavelengths, we plotted the recovered photobleaching rate constants against the relative power E_c_, which measures the amount of light power coupled into the chromophore of each FP (Figure 1B and STAR Methods). Results showed that mEGFP and mCherry outperform (i.e., exhibit smaller photobleaching rate constants than) mGold and mScarlet. This finding is consistent with the literature, which showed the reduced stability of mScarlet relative to mCherry.^31^ However, the low stability of mGold is surprising, given previously published results.^32^

### HaloTag-JF646 outperforms FPs in photostability

The HaloTag protein is a modified haloalkane dehalogenase engineered to covalently bind fluorescent ligands with exceptional photostability and brightness.^13^ Hence, they are excellent candidates for use in FFS-based measurements. However, many quantitative imaging studies continued to use FPs despite their low photostability and the presence of blinking, dark, or low fluorescence states.^33^ In the context of FFS experiments, we surmised that HaloTag might outperform traditional FPs based on the high brightness and photostability of HaloTag ligands.^13^ To optimize the HaloTag-labeling procedure, we constructed a tandem HaloTag-mEGFP plasmid. When the labeling reaction for HaloTag reaches an equilibrium, a stable fluorescence ratio of labeled HaloTag to mEGFP should be expected. Fluorescence labeling of HaloTag reached an equilibrium after 6 h of incubation in 200 nM JF646-HaloTag ligand (Figure 1C). Optimization of HaloTag labeling is crucial for accurately interpreting quantitative measurements of protein oligomerization, as incomplete labeling can lead to a severe underestimation of the actual oligomerization state(s) in PCH analysis. We then compared the photostability of HaloTag-JF646 and mCherry. Under similar excitation power, JF646 remained stable, while 15% mCherry was photobleached within 30 s of illumination in HEK293T cells (Figure 1D).

### PCH resolves molecular brightness in living cells

**Figure 2:**
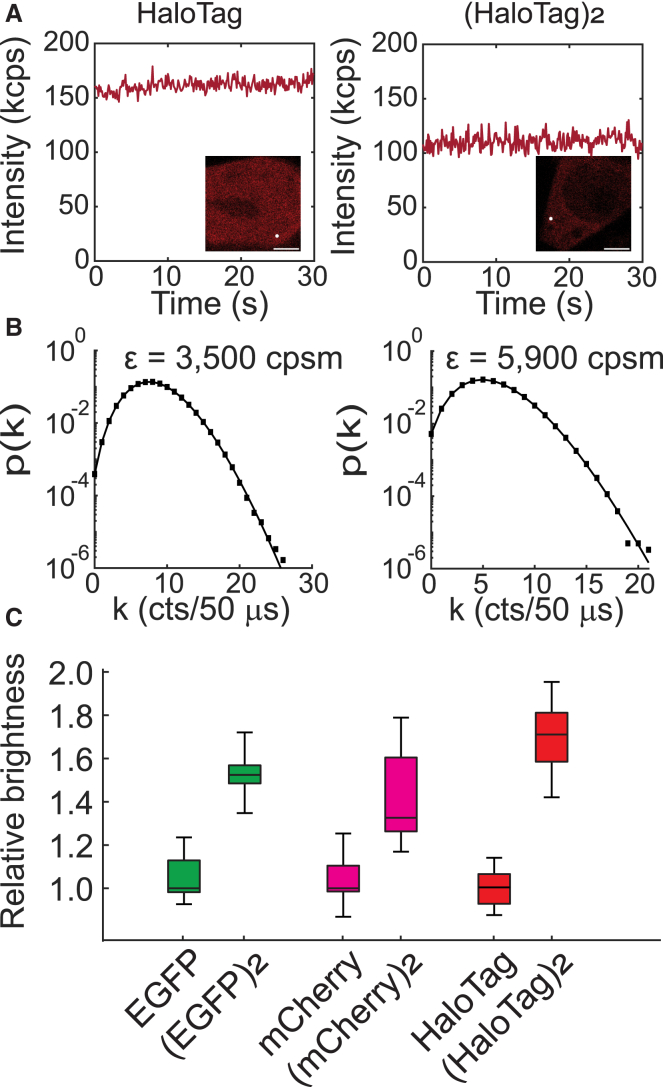
HaloTag-JF646 outperforms fluorescent proteins for quantifying protein oligomerization in living HEK293T cells (A) HEK293T cells expressing a HaloTag monomer (left) or tandem dimer (right) stained with JF646 were imaged using a custom confocal system, and individual spots were selected for PCH analysis (white spot in inset image, scale bars: 6 μm). (B) Fluorescence fluctuations were fit to the PCH model to retrieve brightness in units of counts per second per molecule (cpsm). Black squares represent the data, while the black line shows the fit to the PCH model. (C) The relative molecular brightness of each labeling system (*N* = 10 cells for both EGFP and (EGFP)_2_; *N* = 10 cells for mCherry; *N* = 9 cells for (mCherry)_2_; *N* = 14 cell for HaloTag; *N* = 23 for (HaloTag)_2_). Data are represented as Median ± SD. Also see Figure S2.

To confirm that PCH analysis could resolve the brightness of a monomer from a constitutive dimer, we collected fluorescence fluctuation trajectories in HEK293T cells separately expressing either monomeric or tandem dimeric mEGFP, mCherry, and HaloTag (Figures 2A and 2B). To calculate the molecular brightness for each construct, we employed PCH analysis with a 20 kHz sampling frequency and a detector dead time of 15 ns. Although a dimer should have a molecular brightness twice that of a monomer, various factors, such as protein misfolding, chromophore maturation, and the presence of dark or low-fluorescent states of the chromophore, could result in a lower apparent brightness for the dimer.^34^ As an example, monomeric EGFP is known to occupy a fluorescent state with a probability of 80%.^35^ To compute *p*_*f*_, the probability that each fluorophore is fluorescent, we applied the following model:^34^ $\varepsilon_{n,norm}=\frac{\varepsilon_{n}}{\varepsilon_{1}}=1+{\left(n-1\right)p}_{f}$, where the subscripts represent molecular brightness values of the monomer or an *n*-mer. We used the experimentally determined ratio of the molecular brightness of the dimer and monomer $\frac{\varepsilon_{2}}{\varepsilon_{1}}$ to calculate the probability that each fluorophore is fluorescent. Results showed that the median $\frac{\varepsilon_{2}}{\varepsilon_{1}}$ ratio for mEGFP and mCherry was 1.52 and 1.32, respectively, close to but lower than previously reported values of 1.63 and 1.42 recovered from brightness imaging.^34^ The small differences might result from the use of a different linker sequence that separates the protomers in the tandem dimer constructs, which may affect the folding efficiency of the dimers. Curiously, although the median brightness of mGold is slightly higher than that of mEGFP, both of which were excited at 488 nm, we found no significant difference between the two fluorophores (Figure S1). In contrast, the $\frac{\varepsilon_{2}}{\varepsilon_{1}}$ ratio of HaloTag-JF646 was 1.70, indicating that more HaloTag protein molecules were fluorescent relative to mEGFP or mCherry (Figure 2C) in the tandem constructs. Besides enhanced photostability, the redshifted absorption peak of JF646 allows it to be used as an “orthogonal” label that can be observed without simultaneously activating the commonly used optogenetic proteins. For example, both VfAuLOV and AtCRY2 are activated by 405–488 nm light, overlapping with the absorption spectrum of mEGFP.

### PCH confirms blue light-induced dimerization of VfAuLOV in living cells

**Figure 3:**
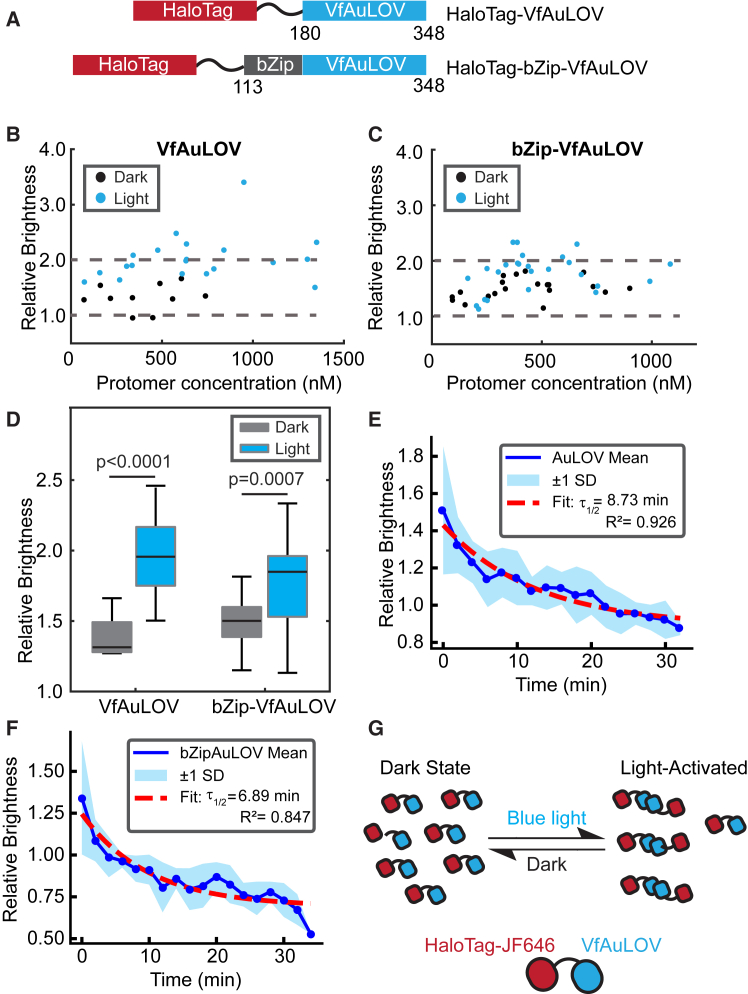
Oligomerization states of VfAuLOV and bZip-VfAuLOV in living HEK293T cells (A) Constructs used in this work. Residue numbering is shown according to the numbering of the full-length proteins. (B) Relative molecular brightness of VfAuLOV (wild-type) in the dark (black) and light-activated (blue) states. Brightness values are relative to the monomeric standard (*N* = 10 cells, dark; *N* = 20 cells, light). (C) Same as (B) but for bZip-VfAuLOV (*N* = 20 cells, dark; *N* = 25 cells, light). In (B) and (C), dashed lines are drawn at the expected brightness of a fully monomeric or dimeric population. (D) The average values of relative brightness for VfAuLOV and bZip-VfAuLOV resolved by PCH. Data are represented as Mean ± SD. (E) Dissociation kinetics of VfAuLOV measured in HEK293T cells overlaid with an exponential fit for the dissociation lifetime (*N* = 10 cells). (F) Same as (E) but for bZip-VfAuLOV (*N* = 5 cells). (G) PCH-inspired model for VfAuLOV in living cells. In the dark state, VfAuLOV exists primarily as monomers. After blue light stimulation, the equilibrium shifts toward a dimeric conformation.

Next, we applied PCH analysis to the VfAuLOV system. Quantification of VfAuLOV’s oligomerization state has so far relied on reconstituted proteins *in vitro*.^18^ It remains unclear whether fusion proteins containing VfAuLOV form primarily dimers or higher-order oligomers in cells in response to blue light stimulation. To quantitatively determine the light-induced oligomerization of VfAuLOV, we transfected HEK293T cells with HaloTag-VfAuLOV and then stained them for 6 h with JF646 HaloTag ligand (Figure 3A). Expression of all constructs used in this study was driven by the ubiquitin C (UBC) promoter, which has a weak promoter strength that measures about 10% of that of the cytomegalovirus (CMV) promoter^36^ (see discussion for the rationale of promoter choice). We first measured the molecular brightness of a population of cells in the dark state and calculated the relative brightness by dividing the absolute brightness by that of the monomeric dye. The relative brightness was then corrected to account for incomplete labeling based on the observed HaloTag dimer:monomer brightness ratio of 1.70.^34^ This correction was applied to all relative brightness values reported for our optogenetic constructs (see STAR Methods for details of brightness correction). Using this procedure, we calculated the corrected relative brightness for VfAuLOV as 1.3 ± 0.2 in the dark (Figure 3B, black dots; Table 1), indicating the existence of a mixed oligomeric population. In a monomer-dimer equilibrium, one can derive that an ε_*app*_ of 1.3 corresponds to a dimer fraction of 18% (see STAR Methods for calculation), indicating residual dimerization of AuLOV-based systems in the dark, consistent with observations in cell-based systems.^16^

**Table 1:** Summary of the relative brightness for each photoactivatable protein variant

Variants	Dark		Light		
	Relative Brightness	Oligomer	Relative Brightness	Oligomer	Dissociation Half-life (min)
VfAuLOV	1.3 ± 0.2	18%	2.0 ± 0.4^a^	100%	8.73
bZip-VfAuLOV	1.5 ± 0.2	33%	1.8 ± 0.3^a^	67%	6.89
AtCRY2	1.1 ± 0.1	<1%	2.8 ± 0.8^a^	27%	4.29
HsCRY2	1.2 ± 0.1	–	1.3 ± 0.2^a^	–	–
AtCRY2(D387A)	1.1 ± 0.1	<1%	1.1 ± 0.2	<1%	–
AtCRY2(R439L)	1.1 ± 0.1	<1%	1.1 ± 0.2	<1%	–
AtCRY2(W374A)	1.5 ± 0.2	5%	2.5 ± 0.5^a^	20%	–

Values are presented as Mean ± SD

The number of cells for each condition is.

*N* = 10 (VfAuLOV, dark), *N* = 20 (VfAuLOV, light).

*N* = 20 (bZip-VfAuLOV, dark), *N* = 25 (bZip-VfAuLOV, light).

*N* = 45 (AtCRY2, dark), *N* = 87 (AtCRY2, light).

*N* = 15 (HsCRY2, dark), *N* = 20 (HsCRY2, light).

*N* = 12 (AtCRY2(D387A), dark), *N* = 12 (AtCRY2(D387A), light).

*N* = 12 (AtCRY2(R439L), dark), *N* = 12 (AtCRY2(R439L, light).

*N* = 20 (AtCRY2(W374A), dark), *N* = 8 (AtCRY2(W374A), light).

Oligomerization percentage is calculated by assuming a monomer-dimer equilibrium model for VfAuLOV variants and a monomer-tetramer equilibrium for AtCRY2 variants.

a The oligomerization state depends on the expression level.

To quantify the absolute protein concentration within living cells, one needs to consider that the brightness value (ε) and number of particles (N) reflect the weighted average of the mixed population. The “apparent” or weighted-average molecular brightness of the two-state model is given by the following equation: $\varepsilon_{app}=\frac{\varepsilon_{1}^{2}N_{1}+\varepsilon_{2}^{2}N_{2}}{\varepsilon_{1}N_{1}+\varepsilon_{2}N_{2}}$, where *ε*_*i*_ and *N*_*i*_(*i* = 1,2) are the brightness and number of particles of the two states.^10^ The fitted value of N_*app*_ reflects not the number of individual protein molecules but the “apparent” number of molecules that correspond to the average brightness value of ε_*app*_. To calculate the true monomeric protein concentration, we note that the following relation holds for all samples: ε_*app*_N_*app*_ = ε_1_N_1_, where the right-hand terms ε_1_ and N_1_ are the monomeric brightness and the number density of monomer equivalents (or protomers), respectively. As ε_*app*_, N_*app*_, and ε_1_ can be experimentally measured, one can derive N_1_, the number of protomers within the excitation focus. Using a standard fluorophore solution, we can calibrate the size of the focal volume and convert the fitted N_1_ into absolute concentration (Figure S2 and STAR Methods). This way, we determined that the intracellular protomer concentration ranges from 50 nM to 1,200 nM (Figure 3B), providing an estimate of the range of expression levels in cells.

Next, we applied 5 min of continuous blue LED light stimulation (6 mW/cm^2^, 450–490 nm, measured at the sample plane) to the same cells on the microscope. The relative brightness appears to show a slight positive correlation with the expression level, particularly between 50 nM and 500 nM (Figure 3B, blue dots). Nonetheless, the average relative brightness of the light-state for VfAuLOV is 2.0 ± 0.4, indicating full conversion to dimers (Table 1). Occasionally, we measured higher relative brightness values (>3) that could indicate higher-order aggregates. Alternatively, higher brightness values could arise from cell movement during the measurement, which could periodically shift the observation volume into a different area with a different local concentration, artificially increasing the observed intensity fluctuations.

When basic residues and a leucine zipper domain were included (bZip-VfAuLOV), the base-level oligomerization increased in the dark, 1.5 ± 0.2 (Table 1), which we attributed to the stabilizing effect of the basic/zipper region. Like VfAuLOV, bZip-VfAuLOV displays light-induced dimerization (Figure 3C), and larger oligomers tend to appear at higher protomer concentration, with an average brightness of 1.8 ± 0.3 across all concentrations (Table 1). The dark-state average of 1.5 corresponds to a dimer fraction of 33%, and the light-state average of 1.8 corresponds to a dimer fraction of 67%. Significant differences were found for the average relative brightness of the dark and light states for both VfAuLOV and Vf-bZipAuLOV (Figure 3D).

We further characterized the dissociation kinetics for both VfAuLOV and bZip-VfAuLOV. HEK293T cells were transfected as above. After a 5-min exposure to blue light, the molecular brightness was immediately measured at the indicated times following the turn-off of the blue light. Each measurement lasted 30 s. The relative brightness values were calculated by normalization with the monomeric fluorophore brightness; all curves were then averaged and fitted with a single-exponential decay: *f* = *Ae*^-*t*/*τ*^+*C*, where *A* is the amplitude, *C* is the base level, and *τ* is the dissociation lifetime, the time it takes for the relative brightness to decay to 1/e. The dissociation half-life *τ*_1/2_, the decay time to 50% of the initial value is calculated as *τ*_1/2_ = *ln*2∗*τ*. Both constructs underwent reversible dimerization with dissociation half-life of 8.73 min for VfAuLOV and 6.89 min for bZip-AuLOV (Figures 3E–3G).

### AtCRY2 and HsCRY2 show distinct oligomerization behavior in living cells

**Figure 4:**
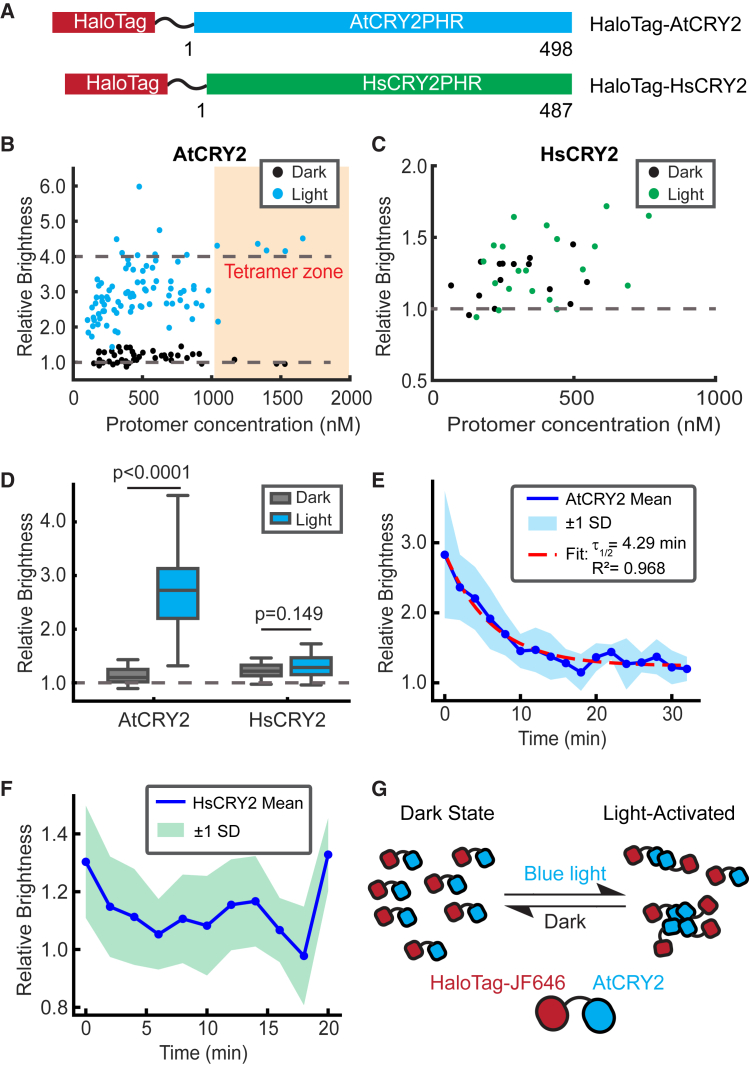
Oligomerization states of AtCRY2 and HsCRY2 in living HEK293T cells (A) Constructs used in this work. Residue numbering is shown according to the numbering of the full-length proteins. (B) Relative molecular brightness of AtCRY2 (wild-type) in the dark (black) and light-activated (blue) states. Brightness values are relative to the monomeric standard. Dash lines are drawn at the expected brightness of a fully monomeric or tetrameric population (*N* = 45 cells, dark; *N* = 87 cells, light). (C) Same as (B) but for HsCRY2. Dashed lines are drawn at the expected brightness of a fully monomeric population (*N* = 15 cells, dark; *N* = 20 cells, light). (D) The average values of relative brightness for AtCRY2 and HsCRY2 resolved by PCH. Data are represented as Mean ± SD. (E) Dissociation kinetics of AtCRY2 measured in HEK293T cells overlaid with an exponential fit for the dissociation lifetime (*N* = 9 cells). (F) Same as (E) but for HsCRY2 (*N* = 7 cells). (G) PCH-inspired model for AtCRY2 in living cells. In the dark state, AtCRY2 exists primarily as monomers. After blue light stimulation, the equilibrium shifts toward a higher-order oligomerization state.

We next set to compare the oligomerization states between AtCRY2 and HsCRY2 to address the distinct behavior between plant and animal CRY2. Previous work showed that AtCRY2 undergoes light-dependent oligomerization, whereas HsCRY2 forms homo-oligomers independent of light.^30^ HaloTag was fused to the N-terminus of AtCRY2 or HsCRY2, and constructs were transfected in HEK293T cells (Figure 4A). For AtCRY2, the average dark-state brightness across all concentrations (up to 1600 nM) is 1.1 ± 0.1 (Table 1), which, although slightly above the baseline of 1.0, would correspond to <1% of tetramers for a monomer-tetramer equilibrium (see STAR Methods for calculation). When considering only data above 500 nM, the average brightness is 1.2, corresponding to 1.8% tetramers (Figure 4B, black dots). We did not attempt to fit a monomer fraction using more complex models (e.g., including dimers) owing to the increased complexity and higher precision required (see discussion). After exposure to blue light, the relative brightness values increased to 2.8 ± 0.8, or 27% tetramer (Figure 4B, blue dots; Table 1). Near-full conversion of AtCRY2 into tetramers was observed in cells expressing more than 1,000 nM proteins.

In contrast, HsCRY2 showed distinct oligomerization responses in HEK293T cells. Animal cryptochromes are primarily photoreceptors that perform both light-dependent and light-independent functions in the regulation of the circadian clock.^37^ For example, human cryptochromes are believed to be a light-independent transcription factor. However, evidence showed that human cryptochrome (HsCRY1) can respond to light and undergo photoreduction in cells. Previous co-immunoprecipitation experiments indicated light-independent oligomerization of HsCRY1 and HsCRY2 in HEK293 cells,^30^ but the stoichiometry of their conformational state is unclear. To define the oligomerization state of human cryptochrome in cells, we applied PCH analysis to HaloTag-HsCRY2. Relative brightness increases at higher concentrations, irrespective of light exposure (Figure 4C). The average relative brightness is 1.2 ± 0.1 in the dark and 1.3 ± 0.2 after light stimulation (Table 1). Unlike AtCRY2, which displayed a significant difference in oligomerization state between light and dark conditions, no such difference was observed for HsCRY2 (Figure 4D). The other difference is the dissociation kinetics. AtCRY2 oligomers rapidly dissociated when blue light was turned off, showing a dissociation half-life of 4.29 min (Figure 4E). In contrast, HsCRY2 oligomers did not change significantly when blue light was turned off, consistent with its light-independent functions (Figure 4F). Taken together, PCH analysis clearly reveals the concentration-dependent, light-inducible oligomerization for AtCRY2 in living cells (Figure 4G).

### AtCRY2 mutants show distinct responses in light-induced oligomerization

**Figure 5:**
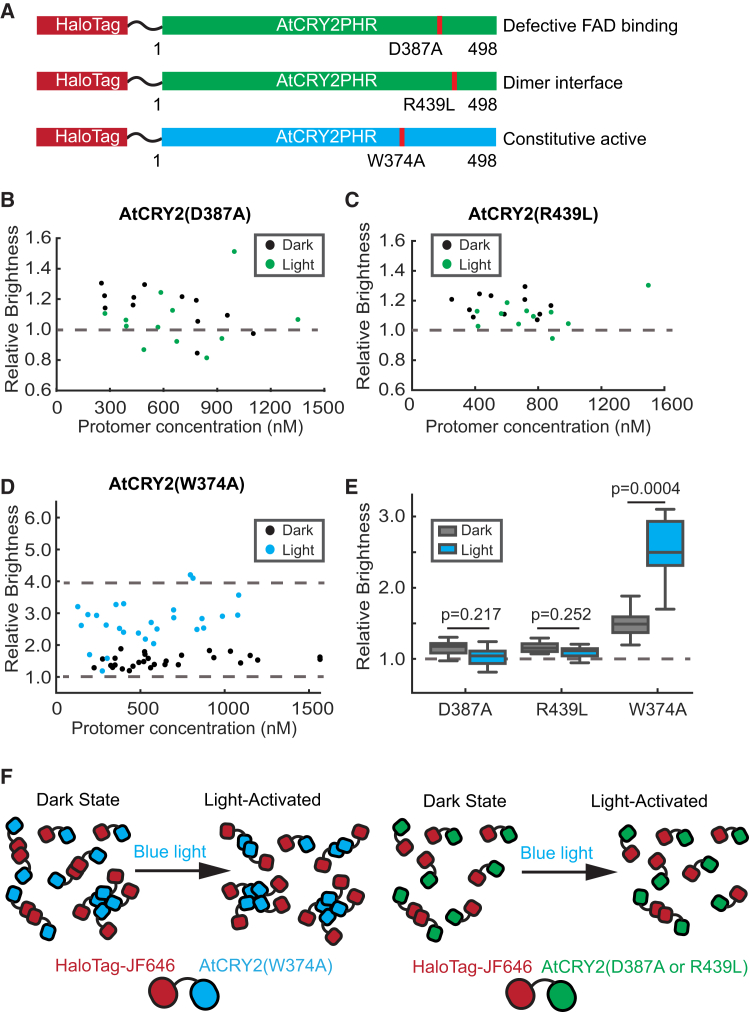
Oligomerization states of AtCRY2 variants in living HEK293T cells (A) Constructs used in this work. Residue numbering is shown according to the numbering of the full-length proteins. (B and C) Relative molecular brightness of AtCRY2(D387A) (B) (*N* = 12 cells, dark; *N* = 12 cells, light) and AtCRY2(R439L) (*N* = 12 cells, dark; *N* = 12 cells, light) (C) in the dark (black) and light-activated (blue) states. Brightness values are relative to the monomeric standard. Dash lines are drawn at the expected brightness of a fully monomeric population. (D) Same as (B) but for AtCRY2(W374A). (*N* = 20 cells, dark; *N* = 8 cells, light) Dash lines are drawn at the expected brightness of a fully monomeric and tetrameric population. (E) The average values of relative brightness for AtCRY2 variants resolved by PCH. Data are represented as Mean ± SD. (F) PCH-inspired model for AtCRY2 variants in living cells. In the dark state, AtCRY2 variants exist primarily as monomers with basal level oligomerization. After blue light stimulation, the equilibrium shifts toward a higher order oligomerization state for AtCRY2(W374A). However, no significant oligomerization is expected to occur for AtCRY2(D387A) or AtCRY2(R439L).

AtCRY2’s light sensitivity renders its wide use in optogenetic applications. As a result, its biochemistry has been better studied, and various oligomerization-modulating mutants have been identified. To further validate PCH analysis and determine the photo response of AtCRY2, we generated three CRY2 mutants, HaloTag-AtCRY2D387A, HaloTag-AtCRY2R439L, and HaloTag-AtCRY2W374A (Figure 5A). The D387A AtCRY2 mutant lacks FAD binding, whereas R439L mutation disrupts the head-to-tail (HT) dimer interface of CRY2.^19^ Both mutants exist mainly as monomers *in vitro*.^23^ PCH analysis showed that the relative brightness for D387A mutant was 1.1 ± 0.1 (dark) and 1.1 ± 0.2 (light), whereas that for R439L was also 1.1 ± 0.1 (dark) and 1.1 ± 0.2 (light), indicating that both mutants mainly existed as monomers (<1% tetramer) in cells within the range of the tested expression level (Figures 5B and 5C; Table 1).

Next, we applied PCH analysis to study AtCRY2(W374A), a constitutively active mutant in plants, even in darkness.^38^ This mutant has been observed to form dimers and tetramers *in vitro*.^19^ Interestingly, the relative brightness of the W374A mutant was only 1.5 ± 0.2 in the dark, indicating its less than full conversion to either dimers or tetramers (Figure 5D, black dots; Table 1). Unexpectedly, the W374A mutant still underwent light-induced oligomerization (Figure 5D, blue dots), with an average brightness of 2.5 ± 0.5, corresponding to approximately 20% tetramerization (Table 1). Statistical analysis revealed a significant difference in oligomerization states for W374A, but not for D387A or R439L (Figure 5E). This is surprising given that the W374A mutation severely reduces photoreduction *in vitro*.^38^ However, the authors of the same study also noted that the W374A mutant still responds to changes in light fluency. Thus, we conclude that the W374A mutant has a higher basal oligomerization state and retains the ability to oligomerize further upon light stimulation in cells. Taken together, these results reveal distinct photooligomerization behaviors between AtCRY2 variants (Figure 5F).

### The potency of optogenetically induced lytic cell death through RIPK3 oligomerization correlates with the oligomerization state of VfAuLOV and AtCRY2

**Figure 6:**
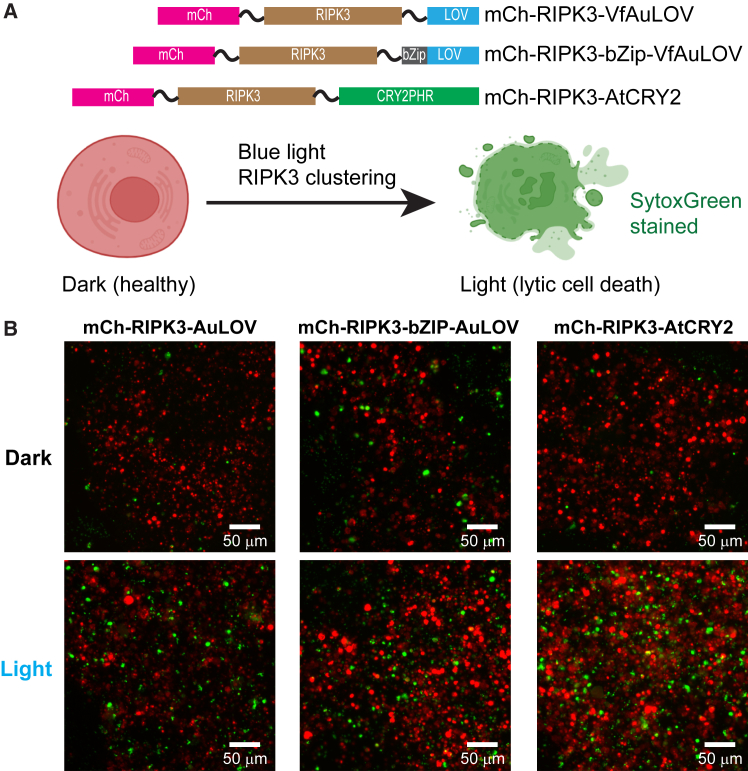
The potency of optogenetically induced lytic cell death depends on oligomerization states (A) (Top) Constructs used in this work. (Bottom) Light-induced clustering of RIPK3 is expected to cause lytic cell death (necroptosis) marked by SytoxGreen staining. (B) HEK293T cells transfected with each construct under dark and blue LED light (465 nm, 0.5 mW/cm^2^, 15 s on/off cycle for 12 h) treatment. Representative Images are from three biological replicates.

To link the oligomerization behavior of photoactivatable proteins with their signaling outcomes, we fused mCherry-RIPK3 (receptor-interacting serine/threonine protein kinase 3) to the N-termini of VfAuLOV, bZip-VfAuLOV, and AtCRY2. RIPK3 clustering leads to lytic cell death, which can be stained by SytoxGreen, a membrane-impermeable nucleic acid staining dye (Figure 6A). We reasoned that the death-inducing potential from each fusion construct differs due to their distinct oligomerization behavior in the dark and light. When expressed individually in cells, the basal level of cell death ranked as bZip-VfAuLOV > VfAuLOV > AtCRY2, which is consistent with their dark oligomerization states. Blue light-induced cell death potential ranked as AtCRY2>bZip-VfAuLOV ∼ VfAuLOV (Figure 6B), suggesting that a higher oligomerization state of RIPK3 can induce more potent cell death, consistent with previous studies.^39^ We would like to emphasize that the photoactivatable proteins, when fused with RIPK3, facilitate the initial association of RIPK3 monomers. However, cell death should result from the functional outcomes of much larger RIPK3 multimers, known as necrosomes, likely through homoassociation mediated by their RIP homology interacting motif domains. Indeed, super-resolution imaging resolved round or rod-shaped necrosomes with a diameter of approximately 45 nm and lengths of hundreds of nanometers for the rod,^40^ which contains homo-oligomers of RIPK3 and RIPK1 that are significantly larger than their monomers. Thus, photoactivatable proteins with a stronger oligomerization potential should initiate RIPK3 homoassociation more readily.

## Discussion

This study characterized the blue-light-induced homotypic protein association of VfAuLOV, AtCRY2, and HsCRY2, which were expressed in HEK293T cells at intracellular concentrations ranging from 50 nM to 1,700 nM and illuminated with mild light power (6 mW/cm^2^ at the sample plane). This light power was chosen because it falls within the range of typical blue light LED output, comparable to the physiological conditions on the sunlight-illuminated Earth’s surface. The blue spectrum of solar irradiance is estimated to be 14.3 mW/cm^2^, assuming 42% of the total solar irradiance (1,361 W/m^2^, as measured by NASA)^41^ is visible light, and the blue spectrum (400–500 nm) accounts for 25% of the visible power. Additionally, this power (6 mW/cm^2^, equivalent to 225 μE m^−2^ s^−1^ or 225 μmol m^−2^ s^−1^ at 450 nm) has been validated by multiple research laboratories, which enables the robust stimulation of various LOV- and cryptochrome-based optogenetic responses *in vitro*^38^^,^^42^ or in cells.^17^^,^^24^^,^^25^^,^^30^^,^^43^^,^^44^^,^^45^^,^^46^^,^^47^ The range of 50–1,700 nM intracellular protein concentration corresponds to the average expression level of the UBC promoter, about 10% of the CMV promoter strength.^36^ The rationale for selecting the UBC promoter was to minimize the side effects of overexpression and to ensure that sufficient fluctuation can be measured in PCH analysis, as higher concentrations imply less relative fluctuation.

Under these conditions, our PCH analysis reveals that variants of VfAuLOV and AtCRY2 share common mechanisms in light-inducible homoassociation but also bear distinct features. The light-inducible oligomerization states of VfAuLOV, bZip-AuLOV, and AtCRY2 highly depend on their intracellular concentration in living cells—the brightness positively correlates with the expression level. Even the light-independent HsCRY2 shows such a positive correlation, indicating that the percentage of high-order oligomers is higher in cells with high expression levels. At intracellular concentration beyond 1,000 nM, VfAuLOV approaches the dimeric state and AtCRY2 approaches the tetrameric state, consistent with biochemical analysis of VfAuLOV and the cryo-EM structure of the tetrameric AtCRY2 structure.^20^ Light-inducible oligomerization appears to be reversible, with a dissociation half-life ranging from 4 to 9 min.

Almost all variants show some base-level oligomerization in the dark (Table 1). Under a two-state model assumption, 18% Vf-AuLOV and 33% bZip-VfAuLOV exist as dimers in the dark. VfAuLOV predominantly forms dimers upon blue light illumination, consistent with structural information gained from the biochemical analysis.^18^ bZip-VfAuLOV has a smaller dynamic range, with less light-inducible oligomerization (67%). This result suggests that VfAuLOV could serve as a better dimerizer in optogenetic applications if a dynamic range is preferred. In contrast, AtCRY2 shows much less basal oligomerization—less than 1% of AtCRY2, AtCRY2(D387A), and AtCRY2(R439L) exist as tetramers in the dark.

Intriguingly, AtCRY2(W374A), a constitutively active mutant with a dimeric and tetrameric structure resolved by cryo-EM in the dark,^19^ shows a slightly increased relative brightness (∼1.5) in PCH analysis up to 1,600 nM, which implies a tetramer population of only 5%. This result suggests that active signaling tetrameric state formation (in the dark) may require an intracellular concentration beyond 1,600 nM. Unexpectedly, W374A still exhibits light-induced oligomerization. Since photoreduction is severely compromised in the W374A mutant, the observed light-induced oligomerization supports the hypothesis that its oligomerization at most partially depends on photoreduction via the Trp-triad (W321, W374, and W397) in AtCRY2. Interestingly, previous studies revealed that AtCRY2 (W374A), despite being constitutively active, still exhibited activity-inhibiting effects on hypocotyl elongation in response to blue light for unknown reasons.^38^ Our PCH result suggests a possible explanation that such a response could result from blue light-induced W374A oligomerization. Inspired by the previous structural study,^19^ we speculated that blue light illumination excites the FAD cofactor, causing a small conformational change that propagates and amplifies larger conformational changes away from the FAD-binding pocket. Such amplified conformational changes, likely near the H-T interface, could facilitate CIB1 binding, as suggested by mutational studies^29^ and cryo-EM structures,^21^ and stabilize the tetrameric state of AtCRY2. Importantly, such a conformational change originating with FAD photoexcitation does not require electron donation from the Trp-triad. Indeed, AtCRY1 (W400F), which lacks an electron donor to FAD, exhibits decent photoreduction in insect cells when exposed to 150 μmol m^−2^ s^−1^ (approximately 4 mW/cm^2^) light at 450 nm.^48^

Since the structure of the AtCRY2 tetramer is readily resolved, we wondered if the same residues and dimer interfaces involved in AtCRY2 self-oligomerization might also play a role in CRY2’s association with other effector molecules in mammals. To test this idea, we first aligned the published structure of FAD-bound *Mus musculus* CRY2 (MmCRY2, PDB: 4I6G), which shares ∼95% amino acid sequence identity with HsCRY2, with the inactive AtCRY2 monomer (PDB: 6K8I) (Figure S3). This alignment showed a moderate root-mean-square deviation (4 Å) but a similar overall fold (Figure S4A). The resulting structural alignment allowed us to identify the residues in MmCRY2 that form a putative HT interface (Figure S4A), which has been previously identified in the oligomeric structure of AtCRY2.^23^ Next, we analyzed the published crystal structure of the CRY-binding domain of murine period circadian regulator (PER) 2 with MmCRY2 PHR^49^ (PDB: 4U8H) and found that the HT interface is partially occluded (Figure S4B). This analysis suggests that the same residues involved in self-oligomerization in AtCRY2 may have evolved to promote substrate binding in mammals, consistent with a model of light-independent signaling in HsCRY2. Our data do not rule out the possibility that light influences the physical interactions of HsCRY2 with other effectors. Whether this is true requires direct experimental testing of light-dependent binding of HsCRY2 to effector molecules, such as PERs, which are known to compete with FAD for binding to MmCRY2.^50^ It is also unknown whether the light absorption of FAD influences FAD binding to HsCRY2, although FAD has been observed to stabilize CRY2 protein in cell culture and mice.^51^

Besides brightness, PCH analysis also directly measures the intracellular concentration of photoactivatable proteins, which may provide insights into the quantitative interpretation of their photooligomerization behaviors. For example, previous work suggests HsCRY2 could homo-associate in a light-independent manner.^30^^,^^52^ Here, PCH analysis indicates that HsCRY2 likely exists as a mixture of monomer and oligomers within a concentration range from 100 to 800 nM. Such a difference could be explained by the fact that a weaker promoter (UBC) was used in this work, compared to the stronger CMV promoter, which is approximately 10 times stronger than UBC, as previously estimated.^36^ Thus, we speculate that complete HsCRY2 homo-association might occur at an intracellular concentration beyond 800 nM in HEK293T cells. Of course, oligomerization is only one potential conformational state for AtCRY2 signaling. A recent study reveals that AtCRY2 functions even in the dark through the binding to effector proteins of Forked-like 1 (FL1) and FL3, and blue light inhibits such interactions.^53^ This further supports the idea that AtCRY2 signaling depends more on its selective binding to effector proteins, either in darkness or light.

Technical advances of this work include a comparative analysis of organic HaloTag ligands and FPs. HaloTag-JF646 outperforms all FPs tested in this work (mEGFP, mCherry, mGold, and mScarlet) in terms of photostability and an improved dimer:monomer molecular brightness ratio, making it a more suitable candidate for PCH analysis. However, caution should be exercised to ensure equilibrated HaloTag labeling. Based on our experience, labeling time is the most critical factor, and a minimum of 6 h is needed for the labeling to reach equilibrium (with a 70% labeling efficiency) in living cells. We emphasize that different batches of chemical labels, even from the same manufacturer, must be tested independently to ensure optimal labeling in the cell line used. The inclusion of self-labeling dyes also invites potential use and optimization of other tags, such as SnapTag (smaller than HaloTag), enabling orthogonal labeling and multiplexed studies in future PCH analysis.

### Limitations of the study

One of the limitations of the current PCH analysis is that only a population average is measured; it does not directly resolve the fraction of monomers, dimers, tetramers, etc., from a mixed population. Although previous work revealed individual monomer and dimer fractions using similar FFS data,^4^ resolving a more complex mixture presents several additional challenges. One problem is that, given a complex mixture, assumptions must be made about the possibility of dimers and trimers in addition to a monomer-tetramer mixture. This means that several models must be tested and selected based on fitting parameters (chi-square). To discriminate between these models, much higher precision would be required to resolve the fractions of monomers, dimers, trimers, and tetramers. This would require much higher laser power, which would limit the maximum observable concentration, and/or longer acquisition times, which would increase the influence of slow cell movements on the data. We also note that acquisition time is *de facto* limited by the dark-state recovery kinetics for photoactivatable proteins used in this work. Even though PCH data acquisition begins immediately after the optogenetic stimulation light is shut off, some photoactivatable proteins may dissociate during the 30-s data acquisition period. We need to shut off the blue LED light during data acquisition to prevent the bleeding through of LED light into the sensitive APDs. As a result, we estimate that the current PCH analysis may underestimate the oligomerization state by 4%–8%, with a dissociation half-life of approximately 4–9 min (Table 1). Future experiments to address these limitations, such as cell fixation during blue light exposure followed by super-resolution imaging, are ongoing in our laboratory.

## Resource availability

### Lead contact

Requests for further information and resources should be directed to and will be fulfilled by the lead contact, Kai Zhang (kaizkaiz@illinois.edu).

### Materials availability

Plasmids generated in this study are in the process of being deposited with Addgene.

### Data and code availability

Data reported in this paper will be shared by the lead contact upon request. PCH fitting was done by the commercial software, VistaVision 64 4.2.584, provided by ISS. This paper does not report additional code. Any additional information required to reanalyze the data reported in this paper is available from the lead contact upon request.

## Acknowledgments

This work is supported by the American Parkinson Disease Postdoctoral Fellowship (award No. 978673) (T.C.). 10.13039/100000057National Institute of General Medical Sciences (R01GM132438) and 10.13039/100000025National Institute of Mental Health (R01MH124827), 10.13039/100000001NSF (award No. 2121003), and an 10.13039/100000001NSF Science and Technology Center for Quantitative Cell Biology grant (award No. 2243257) (K.Z.). We are grateful to Dr. Yuansheng Sun from ISS for insightful comments and support in building the microscope and PCH analysis.

## Author contributions

Conceptualization, T.C. and K.Z.; methodology, T.C. and K.Z.; investigation, T.C., Z.L., Y.L., and T.-J.O; formal analysis, T.C., Z.L., Y.L., and T.J.; visualization, T.C., Z.L., and K.Z.; data curation, T.C. and Z.L.; writing – original draft, T.C. and K.Z.; writing – review & editing, T.C. and K.Z.; funding acquisition, T.C. and K.Z.; resources, K.Z.; supervision, K.Z.

## Declaration of interests

The authors disclose no competing interests.

## STAR★Methods

### Key resources table

**Table:** 

REAGENT or RESOURCE	SOURCE	IDENTIFIER
**Biological samples**		

FastDigest BamHI	Thermo Fisher Scientific	Cat#FD0054
FastDigest BglII	Thermo Fisher Scientific	Cat#FD0083
FastDigest KpnI	Thermo Fisher Scientific	Cat#FD0524
FastDigest HindIII	Thermo Fisher Scientific	Cat#FD0504
FastDigest NotI	Thermo Fisher Scientific	Cat#FD0593
rCutSmart buffer	New England Biolabs	Cat#B6004S
T4 DNA ligase buffer	New England Biolabs	Cat#B0202S
recombinant shrimp alkaline phosphatase	New England Biolabs	Cat#M0371S
T4 DNA ligase	New England Biolabs	Cat#M0202S
Q5 Site-Directed Mutagenesis Kit	New England Biolabs	Cat#E0554S
InFusion HD Cloning Kit	Takara Bio USA, Inc.	Cat#639650
Atto655, free acid	ATTO-TEC GmbH	Cat#AD 655-21
Atto565, free acid	ATTO-TEC GmbH	Cat#AD 565-21
JaneliaFluor 646 HaloTag, 1 nmol	Promega	Cat#HT1060
Dulbecco’s Modified Eagle Medium	Corning	Cat#10-013-CV
Penicillin/streptomycin	Corning	Cat#30-001-CI
Opti-MEM with L-glutamine, no phenol red	Gibco	Cat#11058021
Trypsin + 0.25% EDTA	Gibco	Cat#25200-056
Fetal bovine serum	Avantor	Cat#76419-584
PEI Max	Polysciences	Cat#24765(1)
Glass-bottom 12-well plates, no. 1.5 coverslip	Cellvis	Cat#P12-1.5H-N
Glass-bottom 96-well plates, no. 1.5 coverslip	Cellvis	Cat#P96-1.5H-N
Glass-bottom 35 mm dishes, no. 1.5 coverslip	Mattek	Cat# P35G-1.5-14-C

**Chemicals, peptides, and recombinant proteins**		

Poly-L-lysine	Millipore Sigma	Cat#P1274

**Experimental models: Cell lines**		

HEK293T	ATCC	Cat#CRL-3216

**Oligonucleotides**		

mEGFP.FOR	CACCCAGTCCaagCTGAGCAAAGAC	A206K mutation to build monomeric EGFP
mEGFP.REV	CTCAGGTAGTGGTTGTCG	A206K mutation to build monomeric EGFP
1X-FP.FOR	TCTTTTTGCAGGATCCgccaccatggtgagc	Build fluorescent protein monomer plasmids
1X-FP.REV	CGAATCGATGGGATCTTAAGATCT cttgtacagctcgtccatgcc	Build fluorescent protein monomer plasmids
1X-HT.FOR	TCTTTTTGCAGGATCCgccaccatgGCA GAAATCGGTACTGGC	Build HaloTag monomer plasmid
1X-HT.REV	CGAATCGATGGGATCttaagatct AGTGGTTGGCTCGCC	Build HaloTag monomer plasmid
HT-Linker.FOR	ATGATCGGATCCGGAGGT GGAAGCGGTGGAGGC TCAGCAGAAATCGGT ACTGGCTTTCC	Add flexible linker to HaloTag tandem dimer
HsCRY-Insert.FOR	GGAGGCTCAGAATTCatggcggcaactgtggca	Amplify Human CRY2 PHR insert
HsCRY2-Insert.REV	AGGCCTTGAATTtcaccgtgggtagtccacacca	Amplify Human CRY2 PHR insert
HsCRY2-Vector.FOR	tgaAATTCAAGGCCTCTCGAGC	Amplify pUBC-HaloTag plasmid vector
HsCRY2-Vector.REV	GAATTCTGAGCCTCCACCGC	Amplify pUBC-HaloTag plasmid vector
RemoveRS.FOR	TGAAATTCAAGGCCTCTC	Remove leftover restriction enzyme recognition sequence
RemoveRS-AuLOV.REV	TTTGCGTCTCAGCATATTG	Remove restriction sequence from LOV-based plasmids
RemoveRS-CRY2.REV	ggcagcaccgatcataatc	Remove restriction sequence from CRY2 plasmids
Nterm-HT-Linker.FOR	ggtggaggctcaGAATTCAAGGCCTCTCGAG	Add flexible linker to HaloTag
Nterm-HT-Linker.REV	gcttccacctccAGTGGTTGGCTCGCC	Add flexible linker to HaloTag
Cterm-AuLOV.FOR	CCATCGATTCGAATTCGCCCAG AGCTCACCAGAG	Generate AuLOV template with EcoRI site
Cterm-bZipAuLOV.FOR	CCATCGATTCGAATTCGGTAGTATAAG CTCAGAACTTACTGAAG	Generate bZipAuLOV template with EcoRI site
Cterm-LOV.REV	GAGAGGCCTTGAATTTTAGCTAGCTTT GCGTCTCAGCATATTGGAGG	Generate LOV sequences with EcoRI site
Cterm-CRY2.FOR	CCATCGATTCGAATTCatgaagatg gacaaaaagaccatcgt	Generate CRY2 sequence with EcoRI site
Cterm-CRY2.REV	GAGAGGCCTTGAATTtcagctagcg gcagcaccgatcataat	Generate CRY2 sequence with EcoRI site

**Recombinant DNA**		

pCaggs-mGold	Addgene	#157996
pHR-hSyn-EGFP	Addgene	#114215
pmScarlet_C1	Addgene	#85042
mCherry-CRY2clust	Addgene	#105624
UBC-EGFP	Addgene	#169746
pUBC-1X-HaloTag	This study	N/A
pUBC-2X-HaloTag	This study	N/A
pCMV-1X-mEGFP	This study	N/A
pCMV-2X-mEGFP	This study	N/A
pCMV-1X-mCherry	This study	N/A
pCMV-2X-mCherry	This study	N/A
pCMV-1X-mScarlet	This study	N/A
pCMV-1X-mGold	This study	N/A
pUBC-HaloTag-AuLOV	This study	N/A
pUBC-HaloTag-bZipAuLOV	This study	N/A
pUBC-HaloTag-AtCRY2	This study	N/A
pUBC-HaloTag-AtCRY2(W374A)	This study	N/A
pUBC-HaloTag-AtCRY2(D387A)	This study	N/A
pUBC-HaloTag-AtCRY2(R439L)	This study	N/A
pUBC-HaloTag-HsCRY2	This study	N/A
mCh-RIPK3-AuLOV	Oh et al.^39^	N/A
mCh-RIPK3-bZipAuLOV	Oh et al.^39^	N/A
mCh-RIPK3-AtCRY2	Oh et al.^39^	N/A
HaloTag	Gift from Dr. Jason D. Shepherd, University of Utah	N/A

**Software and algorithms**		

VistaVision	ISS Inc. Version (64) 4.2.584	https://iss.com/
MATLAB R2024b	MathWorks Inc.	https://www.mathworks.com/products/matlab.html
UCSF ChimeraX	Meng et al.^54^	https://www.rbvi.ucsf.edu/chimerax/
Jalview	Waterhouse et al.^55^	https://www.jalview.org/
Fiji	Schindelin et al.^56^	https://imagej.nih.gov/ij/
Python	Version 3.12.7	https://www.python.org/
SciPy	Virtanen et al.^57^	
Matplotlib	Hunter et al.^58^	
Scikit-learn	Pedregosa et al.^59^	

### Experimental model and study participant details

#### Cell culture

Human embryonic kidney cells (HEK293T, ATCC #CRL-3216) were used for all experiments. Cells were maintained in growth medium (DMEM supplemented with 10% FBS and 100 U/mL penicillin, 100 μg/mL streptomycin). Cells were passaged by trypsin digestion and reseeded at 10-40% density. For imaging experiments, cells were seeded in glass-bottom (no. 1.5 coverslip) 12-well tissue culture plates (Cellvis) or glass-bottom 35 mm dishes with a 14-mm coverslip diameter (Mattek). The plates were coated with poly-L-lysine for at least 30 minutes prior to seeding cells. For light-induced cell death, HEK293T cells were seeded in a 48-well plate with 300 μL of Dulbecco’s Modified Eagle Medium (DMEM) supplemented with 10% fetal bovine serum (FBS) and penicillin-streptomycin. Cells were incubated at 37°C with 5% CO_2_ for 24 hours before transfection.

Cells were transfected at 40-60% confluence with PEI Max using 0.5-1 μg of DNA per well/dish. In some experiments, protein expression levels were lowered further by diluting plasmid DNA containing our constructs with empty pCS2+ plasmid DNA. After optimization, we found that 50 ng of construct DNA + 450 ng of empty pCS2+ plasmid DNA provided optimal expression levels without reducing transfection efficiency too much. PEI:DNA ratio was kept at 3:1 (v/w) and mixed with 60 μL (when using 35 mm dishes) or 100 μL (when using glass-bottom 12-well plates) of DMEM per well. After thoroughly mixing the transfection solution and incubating at room temperature for at least 15 minutes, the solution was added to the cells drop by drop. Cells were placed in the incubator for 4 hours, after which the medium was changed. Before imaging, the growth medium was replaced with imaging medium. Imaging medium was prepared by mixing Opti-MEM with L-glutamine (cat # 11058021, Gibco) with 10% fetal bovine serum and antibiotics as above. Imaging medium contained no phenol red. When cells remained out of the incubator for several hours for imaging, 25 mM HEPES, pH 7.4 (cat # 25-060-CI from Corning) was added to the imaging medium. Cells were kept at ambient temperature for no more than 3 hours. For light-induced cell death, 0.3 μg of optogenetic RIPK3-encoding plasmids were complexed with 0.9 μg of PEI MAX in serum-free DMEM to a final volume of 30 μL. The transfection mixture was added dropwise to the center of each well and incubated for 4 hours, after which the medium was replaced with complete DMEM to minimize cytotoxicity. At 20 hours post-transfection, the medium was replaced again to remove spontaneously dead cells while preserving viable cells.

### Method details

#### Plasmid construction

Plasmids were generated by InFusion assembly or restriction digest. Plasmids containing only monomeric fluorescent proteins/HaloTag were generated using InFusion assembly. The pCS2+ vector backbone with cytomegalovirus (CMV) promoter was linearized by digesting with BamHI, and the inserts were amplified with overlap extension PCR to generate complementary overhangs. The insert and vector were combined with the InFusion HD Enzyme premix contained within the InFusion HD Cloning Kit. The monomer plasmids contained a BamHI site before the Kozak sequence, a BglII site after the coding sequence but before the stop codon, and a KpnI site after the stop codon. To generate plasmids containing tandem oligomers, the monomer plasmids were digested with either BglII/KpnI (to generate the new vector backbone) or BamHI/KpnI (to generate the new insert). To generate tandem oligomer plasmids with the flexible glycine/serine linker, the second gene sequence was amplified with PCR using the monomeric plasmid as a template, with the linker and BamHI restriction site added via primer extension. The PCR product was then digested with BamHI/KpnI. The monomer was digested with BglII/KpnI and ligated with the digested PCR product. Phosphates were removed from each vector with rSAP prior to ligation. T4 DNA ligase was used for all ligation reactions. Fusions of HaloTag with VfAuLOV and AtCRY2 variants were assembled with restriction digest cloning using templates that had previously been generated using InFusion cloning. The vector plasmid contained the HaloTag sequence followed by the flexible linker/EcoRI site before the stop codon, and a KpnI site after the stop codon. The insert plasmids contained an N-terminal EcoRI site followed by either the LOV or CRY2 sequences, a stop codon, and a KpnI site. Plasmids were double-digested with EcoRI and KpnI and ligated. Colonies were screened with restriction digest or colony PCR, and the final product was confirmed by sequencing. For longer, repetitive sequences such as tandem oligomers, we used the Plasmidsaurus sequencing service.

For FFS experiments, we often found that the CMV promoter produced very high protein concentrations that are not compatible with FFS. For this reason, we generated plasmids with the Ubiquitin C promoter controlling gene expression of our constructs, which express proteins constitutively but at lower levels. These plasmids were generated by digesting the pCS2-CMV plasmids with HindIII/NotI (which are placed before and after the gene sequence) to generate the insert. The insert was ligated into the vector, which was generated by digesting UBC-EGFP (Addgene #169746) with HindIII/NotI. The cloning proceeded as above for other ligation reactions. Site-directed mutagenesis was further performed on the final HaloTag-VfAuLOV/AtCRY2 plasmids to remove trailing residues on the C-terminus (left over by restriction digestion used to build the templates). The final constructs for all plasmids containing HaloTag are in the process of being deposited with Addgene. Information on plasmids and primers used in this work is shown in the key resources table.

#### Fluorescence confocal imaging

Data presented in Figures 1A and 1B were collected using an LSM 710 laser scanning confocal microscope (Zeiss). All images were obtained using the 40×, 1.2 NA water immersion objective. The excitation was either a 488 nm, 514 nm, or 561 nm laser line. Power measurements were taken at the objective for different settings of the acousto-optic tunable filter that adjusts the laser power on our system. Images were acquired using a pixel dwell time of 12.54 microseconds, 256 × 256 pixels per image, with a pixel size of 100 nm. The pinhole size was always set to 1 Airy unit.

#### Home-built microscope setup

We outfitted our Olympus IX73 microscope (Evident) with a galvanometer-driven scanning mirror unit provided by ISS Inc. (Champaign, IL). To observe transfected cells before FFS imaging, we coupled the laser excitation into the back port of the microscope and illuminated the sample dish in epifluorescence mode. The fluorescence was observed through the eyepiece, and cells of interest were centered by manually moving the translation stage. Alternatively, we used the built-in incandescent light of the IX73 to locate the focal plane containing cells before switching on the laser light for FFS measurements.

To augment the system with confocal scanning, the excitation lasers were split from the epifluorescence light path with a 30/70 beamsplitter (BSS10, Thorlabs). Our system includes three laser lines: 488 nm 100 mW c.w. and 561 nm 50 mW c.w, both Spectra Physics Excelsior models, and a 638 nm 40 mW laser diode (cat #L638P040, ThorLabs) in a mounting system with temperature and drive current control (cat # LTC56A, ThorLabs), with temperature maintained at 25°C. The lasers were then coupled into a single-mode, polarization-maintaining fiber optic patch cable (P5-405BPM-FC-5, Thorlabs) using an achromatic fiber port (cat # PAF2-A4A, ThorLabs) installed in a fiber port adaptor (cat # CP08FP). The fiber delivered the light into the scanning unit (M612, ISS Inc.). The M612 delivered the excitation light through the right-side port of our microscope using either a dual bandpass/longpass dichroic beamsplitter (ZT488/561rpc, Chroma Technology Corporation) or a quadruple bandpass dichroic beamsplitter (405/488/561/635 nm lasers BrightLine® quad-edge laser dichroic beamsplitter, cat # FL-007046, SemRock). The quadruple dichroic filter was used when the 638 nm laser diode was used. The fluorescence was collected along the same path and focused onto an avalanche photodiode (SPCM-AQRH-44-BR2, Excelitas) (APD). Emission filters were placed in front of the APD detector depending on the laser used: 525/50 nm bandpass (cat # ET525/50m, Chroma Technology) for 488 nm, 570 nm long pass (cat # ET570lp, Chroma Technology) for 561 nm, or 640 nm longpass (cat # BLP01-633R-25, Semrock) for 638 nm. The APD was operated in photon-counting mode, with counts sampled by a USB-connected data acquisition card. Data were acquired by storing the photon counts in time-tagged mode, allowing the counts to be binned at lower frequencies during data analysis. The maximum sampling frequency used was 2 MHz. Data was analyzed on a desktop computer using VistaVision (ISS).

#### Photobleaching data acquisition and analysis

The cells were chosen so that, for each power setting, cells of similar intensity were selected for measurement. The PMT gain was varied to place the average intensity in the middle of the detector's dynamic range (∼1000-2000 a.u., out of a maximum of 4096 a.u.). Fifty frames were obtained by unidirectional line scanning using a commercial laser scanning confocal system (LSM 710, Zeiss). For each fluorophore/power combination, three cells were measured. Data were analyzed using Fiji software (ImageJ) and MATLAB (Mathworks). Frames 3-25 were isolated, and the intensity was normalized to the initial intensity. Frame 3 was considered as t = 0 for this analysis. The data were then fit to the function

$$I\left(t\right)=e^{-kt}$$

using a nonlinear least squares approach. Rate constants (*k*) were fit using data from three cells for each fluorophore/power setting, and the average and standard deviation of the rate constants from the three cells are reported in this work, and the relative power *E*_*C*_ is given by

$$E_{C}=P_{\lambda }\times \epsilon_{\lambda }$$

Where *P*_*λ*_ is the incident power at wavelength λ (measured at the objective) and *ϵ*_*λ*_ is the molar extinction coefficient of the FP at wavelength λ. Calculating *E*_*C*_ for each FP used allows us to compare the photostability of FPs that absorb at different wavelengths while accounting for the power output of the laser lines used.

#### Calibration of HaloTag labeling efficiency

HEK293T cells were seeded in a 35 mm plastic dish at 15% confluency and incubated for 20 hours, when cells were transfected with 1 μg HaloTag-mEGFP plasmid with 3 μL PEI transfection reagent in 200 μL of FBS-free DMEM medium. Transfected cells were replated in PLL-coated 96-well plates and incubated overnight. Cells were then stained with 200 nM JF646 for different amounts of time (2, 4, 6, 10, and 24 hours). After the indicated time, cells were destained with DPBS containing Ca^2+^/Mg^2+^. Imaging medium was used to incubate cells during experiments. Fluorescence images were collected using the ImageXpress Pico Automated Cell Imaging System (Molecular Devices), which measures the fluorescence intensity of JF646 and mEGFP in each cell at each time point. A 4.8% imaging area and a 3×3 imaging pattern were used for imaging each well with a 10× objective lens. HaloTag-JF646 was imaged using the Cy5 channel (excitation 610-650 nm, emission 675-720 nm), and mEGFP was imaged using the FITC channel (excitation 445-485 nm, emission 509-539 nm). Images were analyzed with ImageJ to quantify the labeling efficiency. For each cell, the fluorescence intensity ratio was calculated by (integrated density of HaloTag-JF646) / (integrated density of mEGFP). The average ratio from four technical replicates was then calculated for each well and plotted against staining time using MATLAB, with the maximum value normalized to 1.0.

#### Measurement of APD dead time

The relationship between the observed fluorescence intensity, variance, and detector parameters for a constant light source are given by^60^

$$Q={2p}_{a}-2\left\langle k\right\rangle f_{s}\tau_{d}={2p}_{a}-2I\tau_{d}$$

Where *p*_*a*_ is the afterpulsing probability, ⟨*k*⟩ is the average photon count rate in counts per sample period, *f*_*s*_ is the sampling frequency in Hz, *τ*_*d*_ is the detector dead time in seconds, and *I* is the fluorescence intensity in counts per second, equivalent to ⟨*k*⟩×*f*_*s*_. Mandel’s Q parameter is given by

$$Q=\frac{\left\langle {\Delta k}^{2}\right\rangle -\left\langle k\right\rangle }{\left\langle k\right\rangle }$$

Where ⟨*k*⟩ and ⟨Δ*k*^2^⟩ are the average and variance of the fluorescence intensity. To measure the afterpulsing probability and dead time, we measured Mandel’s Q parameter for a solution of highly concentrated dye solution (20 μM of Atto565) using a 561 nm laser line. At this concentration, fluctuations due to molecular diffusion are absent, and the only fluctuations present arise from the detector’s response. To generate a linear fit to the model, we increased the fluorescence intensity by increasing the power of the excitation laser. We calculated Q directly from the fluorescence intensity traces and fit the data to a linear model Q = 2*p*_*a*_-2*Iτ*_*d*_. Since correcting for afterpulsing effects has little effect on the resulting PCH fits,^60^ we only include the dead time in our fitting procedure.

#### Measurement of FFS observation volume

We related the number of particles recovered by PCH to the absolute concentration by independently measuring the observation volume of our microscope using a solution of fluorescent dye with known concentration (Figure S2). We prepared a 20 nM solution of Atto655 (free acid) diluted in MilliQ purified water with 0.05% Tween-20 (v/v). The detergent was included to reduce the adsorption of dye molecules to the surface of the coverslip. The concentration of the dye stock solution was measured independently in the detergent/water solution using absorption spectroscopy with a Cary 3500 UV-Vis Compact Peltier Spectrophotometer (Agilent) and an extinction coefficient of *ε*_655_=125,000*M*^-1^*cm*^-1^. The dye was diluted to 20 nM in a single step. A 400 μL aliquot of the diluted dye solution was added to the same glass-bottom dishes used for cell experiments. The dye was excited using the 638 nm laser line, and the laser power was adjusted until approximately 2 μW/cm^2^ power was observed when measured just before the objective lens. We measured laser power by using a threaded adaptor (cat # SM1BC, ThorLabs) that connects our power meter probe (cat # S121C, ThorLabs) to the microscope turret, allowing for precise and consistent power measurements each day. Fluorescence was collected with a 640 nm longpass emission filter. Single-point FFS data was collected for 30 seconds. PCH data was binned at 20 kHz and fit by fixing the detector dead time to 15 ns and sampling frequency at 20 kHz in the fitting algorithm. For this measurement, the pinhole was set to approximately 1 Airy unit (56 μm for our system with 80× total magnification). All measurements and calculations in this work that refer to absolute concentrations were measured using this pinhole setting.

Fitting the resulting histogram using the PCH model resulted in a recovered number of particles N. To calculate the observation volume, we applied the relation

$$V=\frac{N}{C\times N_{A}}$$

Where V is the observation volume in liters, N is the number of particles measured by PCH, C is the known concentration of the dye solution in molar units, and *N*_*A*_ is Avogadro’s constant. We adjusted the laser power until PCH measurements confirmed consistent PCH fits. We calibrated the system each day to account for any changes in the microscope's collection efficiency function (slight changes in the size of the variable pinhole, temperature effects on alignment, etc.) that might occur from day to day.

#### Fluorescence staining of cells for FFS

For all FCS/PCH experiments, HaloTag fusion constructs were labeled with 200 nM Janelia Fluor 646 HaloTag ligand (Promega, catalog # HT1060) in growth medium for at least 6 hours, followed by two destaining washes with DPBS containing Ca^2+^/Mg^2+^. Imaging medium supplemented with 25 mM HEPES, pH 7.4, was used during imaging experiments, which occurred at ambient temperature (22-23°C).

#### PCH of live cells

All PCH measurements were made using our custom FFS microscope. Fluorescence intensity was measured for either 10 seconds at each spot (fluorescent protein fusion constructs) or 30 seconds at each spot (HaloTag fusion constructs), with at least 6 spots measured for each cell. We fit the binned counts from each spot (20 kHz binning frequency, 15 ns dead time) using PCH. Each trajectory was inspected manually, and trajectories that included significant artifacts due to cell movement were not analyzed. When measuring HaloTag fusion constructs, small intensity drifts (<10% change over the entire trajectory) were occasionally identified, and analysis was restricted to ≥ 10-second regions where no intensity drift was observed. The brightness of each cell was calculated as the average of the brightness values from each spot to calculate the average molecular brightness. Any individual spot with a brightness that differed from the average by more than 1.5σ, where σ represents the sample standard deviation, was excluded from the final analysis.

For light activation, the entire dish was exposed to blue light for 5 minutes (∼ 6 mW/cm^2^ at the sample plane). Power density was measured using a power meter (cat # PM100D, ThorLabs). Immediately after turning off the blue light, a single 30-second measurement was taken for each cell to minimize the effect of dark state recovery during the measurement.

#### Calculation of protomer concentration

For light-exposed cells, the HaloTag fusion constructs are expected to exist as a population of mixed oligomers. To calculate the protomer concentration in cells, we used the following equation

$$\varepsilon_{app}N_{app}=\varepsilon_{1}N_{1}$$

Thus, one can calculate a “protomer equivalent” concentration for a sample of arbitrary brightness by dividing the total count rate by the monomeric brightness. After performing this calculation, we convert it to absolute concentration using the calibrated measurement of the observation volume (1.02 fL). Finally, we divide by the labeling efficiency (70%) to account for unlabeled molecules. Our protomer concentration, therefore, represents the expected concentration of all (labeled + unlabeled) protomers in the cell at the measurement location. These corrections were made before adjusting the molecular brightness into relative units.

#### Correction of relative brightness values

For all measurements reported in Figures 3, 4, and 5, we normalized the molecular brightness values to the monomeric standard and corrected the relative molecular brightness values to account for incomplete labeling, thereby simplifying the interpretation of the scatter plots. We used two equations to correct the measured brightness ε_*app*_. First, we normalize the brightness to the monomeric brightness:

εnorm=εappε1

If ε_*app*_≤ε_1_ (due to experimental uncertainty or noise), we use ε_*norm*_ directly as the corrected relative brightness. If ε_*app*_>ε_1_, then we use the following correction^34^

$$\varepsilon_{norm,corrected}=1+\frac{\varepsilon_{norm}-1}{p_{f}}$$

Where *p*_*f*_ is the fluorescence probability determined by our calibration measurement of the HaloTag-JF646 tandem dimer and monomer (0.7 for this work). After these corrections, a relative brightness value of 2 corresponds to the relative brightness expected for a constitutive dimer. This can be seen by substituting our measured (uncorrected) brightness ratio of ε_*norm*_=1.7 for the HaloTag-JF646 dimer, and the corresponding fluorescence probability *p*_*f*_=0.7:

$$\varepsilon_{norm,corrected}=1+\frac{1.7-1}{0.7}=1+1=2$$

#### Calculation of oligomerization percentage from the relative brightness based on a two-state model

For VfAuLOV and bZip-VfAuLOV, a monomer-dimer equilibrium model is used. Assuming the fraction of dimer is x

$$\varepsilon_{app}=\frac{\varepsilon_{1}^{2}*N_{1}+\varepsilon_{2}^{2}*N_{2}}{\varepsilon_{1}*N_{1}+\varepsilon_{2}*N_{2}}=\frac{\varepsilon_{1}^{2}*\left(1-x\right)+\varepsilon_{2}^{2}*x}{\varepsilon_{1}*\left(1-x\right)+\varepsilon_{2}*x}=\varepsilon_{1}*\frac{\left(1-x\right)+{\left(\frac{\varepsilon_{2}}{\varepsilon_{1}}\right)}^{2}*x}{\left(1-x\right)+\frac{\varepsilon_{2}}{\varepsilon_{1}}*x}$$

Thus, the corrected, normalized brightness

$$\frac{\varepsilon_{app}}{\varepsilon_{1}}=\frac{\left(1-x\right)+{\left(\frac{\varepsilon_{2}}{\varepsilon_{1}}\right)}^{2}*x}{\left(1-x\right)+\frac{\varepsilon_{2}}{\varepsilon_{1}}*x}=\frac{3x+1}{x+1}$$

In the last equation, we noted that the dimer:monomer ratio $\frac{\varepsilon_{2}}{\varepsilon_{1}}=2$, Thus, a relative brightness of 1.5 suggests $\frac{3x+1}{x+1}=1.5$, or *x*=1/3. A pure dimeric population would indicate *x*=1 or $\frac{\varepsilon_{app}}{\varepsilon_{1}}=\frac{3+1}{1+1}=2$.

For AtCRY2 and variants, a monomer-tetramer equilibrium model is used. Assuming the fraction of tetramer is x, we can similarly get

$$\frac{\varepsilon_{app}}{\varepsilon_{1}}=\frac{\left(1-x\right)+{\left(\frac{\varepsilon_{4}}{\varepsilon_{1}}\right)}^{2}*x}{\left(1-x\right)+\frac{\varepsilon_{4}}{\varepsilon_{1}}*x}=\frac{15x+1}{3x+1}$$

Thus, a relative brightness of 1.1 suggests $\frac{15x+1}{3x+1}=1.1$, or *x*=0.0085. A pure tetrameric population would indicate *x*=1 or $\frac{\varepsilon_{app}}{\varepsilon_{1}}=\frac{15+1}{3+1}=4$.

#### Light-induced lytic cell death

Optogenetic activation was carried out using a custom-built blue LED system (465 nm, 0.5 mW/cm^2^, 15 s on/off cycle) for 12 hours. Plasma membrane rupture was assessed by staining cells with 250 nM SytoxGreen, and fluorescence imaging was performed using a Leica DMI8 fluorescence microscope with a 10× objective lens to visualize SytoxGreen (GFP channel, excitation 450-490 nm, emission 500-550 nm) and transgene expression (TXR channel, excitation 540-580 nm, emission 592-668 nm).

#### Structural model analysis

Molecular graphics and analyses shown in Figure S4 were performed with UCSF ChimeraX, developed by the Resource for Biocomputing, Visualization, and Informatics at the University of California, San Francisco, with support from National Institutes of Health R01-GM129325 and the Office of Cyber Infrastructure and Computational Biology, National Institute of Allergy and Infectious Diseases.

Structural alignment of AtCRY2 and MmCRY2 was done using the “Matchmaker” tool in ChimeraX using C^α^ atoms. The Needleman-Wunsch alignment algorithm was used with the BLOSUM-62 substitution matrix. Secondary structure weighting was set to 0.3. The intra-helix gap opening penalty and the intra-strand gap opening penalty were set to 18. All other gaps were assigned a penalty of 6. The resulting amino acid sequence alignment was displayed in Figure S3 using Jalview and was used to identify residues on MmCRY2 that correspond to the HT interface residues of AtCRY2.

### Quantification and statistical analysis

To compare the brightness of each fluorophore under different experimental conditions (e.g., dark vs. light), we performed unpaired two-tailed t-tests with Welch’s correction for unequal variances. This test was selected because the number of measurements and the variance of brightness values differed between the light and dark groups for several constructs. The statistical tests were conducted separately for each fluorophore, comparing the dark and light conditions. A threshold of p < 0.05 was used to determine statistical significance, although exact values are provided for transparency. All analyses and data visualizations were performed using Python (Matplotlib, Seaborn, Pandas, and SciPy libraries).

#### Dissociation kinetics curve fitting

Fluorescence dissociation kinetics were analyzed by fitting the mean signal intensity over time to a single-exponential decay function of the form: *B*(*t*)=*Ae*^-*t*/*τ*^+*C*, where *B*(*t*) is the relative brightness at time t, *A* is the amplitude, *τ* is the decay time constant, *C* is the residual signal at long times. The dissociation half-life is calculated as *τ*_1/2_=*ln*2∗*τ*. For each condition, replicate measurements were averaged at each time point, and the standard deviation was calculated to represent variability. Fitting was performed using nonlinear least squares regression via the curve_fit function in the scipy.optimize module (Python, version 3.12.7), which minimizes the sum of squared residuals between experimental data and the model. Initial parameter guesses were chosen empirically to aid convergence. Fits were evaluated visually and numerically by calculating the coefficient of determination R^2^, using the r2_score function from sklearn.metrics. An R^2^ value close to 1 indicates a strong linear relationship between concentration and brightness.
