# Supplementary material for: Resolving oligomeric states of photoactivatable proteins in living cells via photon counting histogram analysis
Source: iScience. 2025 Oct 23;28(11):113848. doi: 10.1016/j.isci.2025.113848 (PMC12661439; doi:10.1016/j.isci.2025.113848)
Supplement: Document S1. Figures S1–S4 [file mmc1.pdf]

iScience, Volume *28*

Supplemental information

Resolving oligomeric states of photoactivatable proteins in living cells via photon counting histogram analysis

Tyler Camp, Zixiao Li, Yushan Li, Teak-Jung Oh, and Kai Zhang

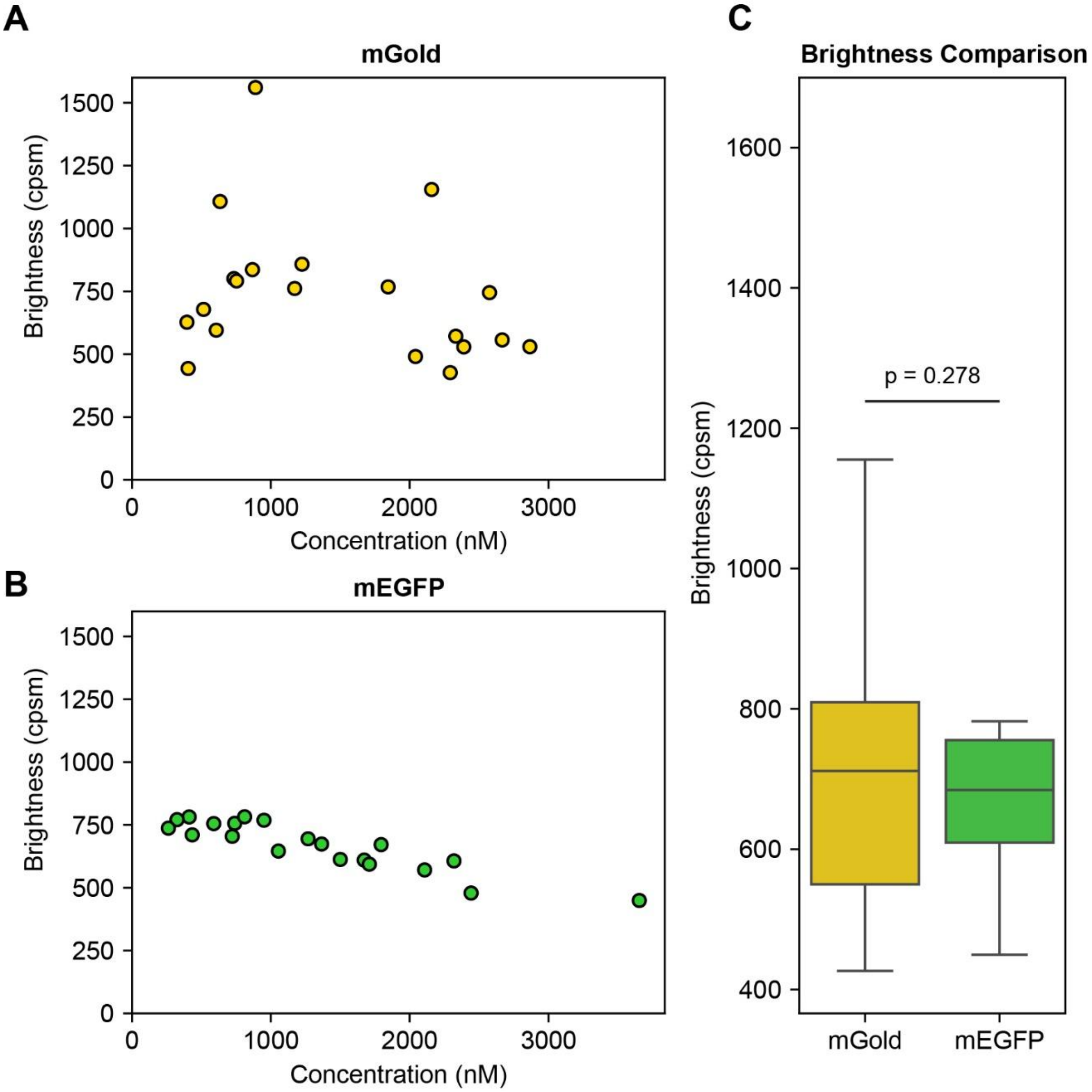

Figure S1. Comparison of molecular brightness between mGold and mEGFP. Brightness of fluorophores, related to Figure 1. Brightness of **(A)** mGold (N=20 cells) (and **(B)** mEGFP (N=20 cells) in HEK293T cells. **(C)** Boxplot of the median and 25%-75% range of brightness between mGold and mEGFP. Data are represented as Mean ± SD.

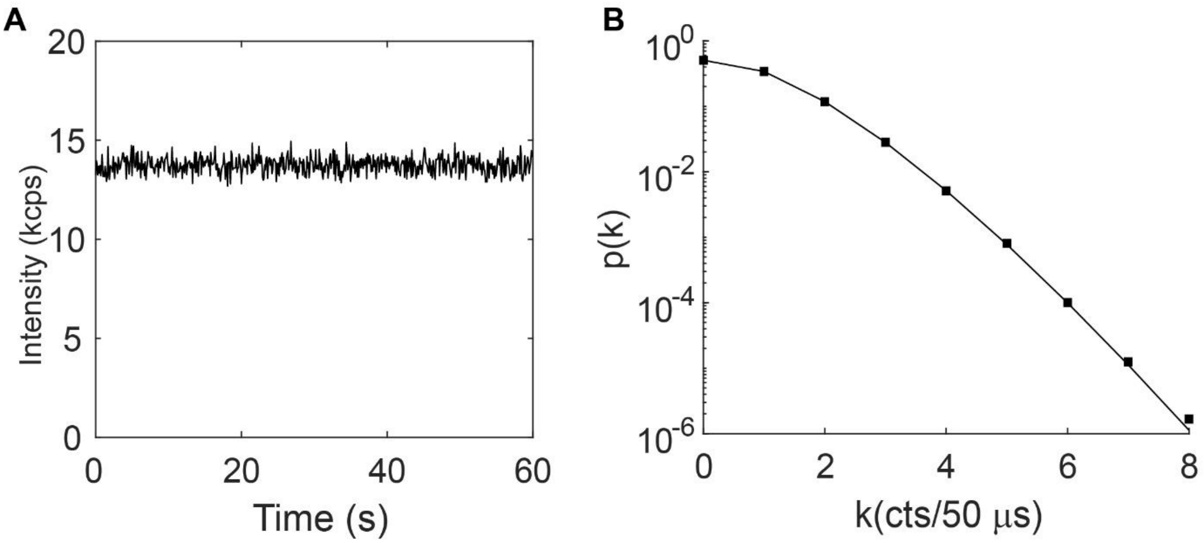

Figure S2. Representative calibration standard for PCH measurements. Fluorescence intensity trajectory and photon counting histogram, related to Figure 2. A solution of 20 nM Atto655 in Milli-Q water + 0.05% Tween-20 (v/v) was measured at a single position within the sample above the coverslip (A) and fit to the PCH model (B). PCH data is shown as black squares, with the model fit drawn as a black line. For this sample, the molecular brightness was 1,200 counts per second per molecule (cpsm), the number of particles was 12, and the chi-square value was 0.69.

```
AtCRY2    1   ---------------MKMD-KKTIVWFRRDLRIEDNPALAAAAHEG-SVFPVF
MmCRY2    1   MAAAAVVAATVPAQSMGADGASSVHWFRKGLRLHDNPALLAAVRGARCVRCVY

AtCRY2    37  IWCPEEEGQFYPGRASRWWMKQSLAHLSQSLKALGSDLTLIQTHNTISAILDC
MmCRY2    54  ILDPWFAASSSVGINRWRFLLQSLEDLDTSLRKLNSRLFVVR-GQPADVFPRL

AtCRY2    90  IRVTGPTKVVFNHLYDPVSLVRDHTVKEKLVERGISVQSYNGDLLYEPWEIYC
MmCRY2    106 FKEWGVTRLTFEYDSEPFGKERDAAIMKMAKEAGVEVVTENSHTLYDLDRIIE

AtCRY2    143 EKG-KPFTSFNSYWKKCLDMSI-ESVMLPPPWRLMPITAAA------EAIWAC
MmCRY2    159 LNGQKPPLTYKRFQALISRMELPKKPAVAVSSQQMESCRAEIQENHDDTYGVP

AtCRY2    188 SIEELGLENEAEKPSNALLTRAWSPGWSNADKLLNEFIEKQLIDYAKNSKKVV
MmCRY2    212 SLEELGFPTEGLGPA------VWQGGETEALARLDKHLERKA--WVANYERPR

AtCRY2    241 GN------STSLLSPYLHFGEISVRHVFQCARMKQIIWARDKNSEGEESADLF
MmCRY2    257 MNANSLLASPTGLSPYLRFGCLS--CRLFYYRLWDLYKKVKRNS---TPPLSL

AtCRY2    288 LRGIGLREYSRYICFNFP-FTHEQSLLSHLRFFPWDADVDKFKAWRQGRTGYP
MmCRY2    305 FGQLLWREFFYTAATNNPRFDRMEGNPICIQ-IPWDRNPEALAKWAEGKTGFP

AtCRY2    340 LVDAGMRELWATGWMHNRIRVIVSSFGVKF-LLLPWKWGMKYFWDTLLDADLE
MmCRY2    357 WIDAIMTQLRQEGWIHHLARHAVACFLTRGDLWVSWESGVRVFDELLLDADFS

AtCRY2    392 CDILGWQYISGSIPDGHELDRLDNPALQGAKYDPEGEYIRQWLPELARLPTEW
MmCRY2    410 VNAGSWMWLSCS-AFFQQFFHCYCPVGFGRRTDPSGDYIRRYLPKLKGFPSRY

AtCRY2    445 IHHPWDAPLTVLKASGVELGTNYAKPIVD-IDTARELLAK
MmCRY2    462 IYEPWNAPESVQKAAKCIIGVDYPRPIVNHAETSRLNIER
```

Figure S3. Structural alignment of AtCRY2 and MmCRY2. The dark-state monomer of AtCRY2 (PDB: 6K8I) was aligned to the structure of FAD-bound MmCRY2 (PDB: 4I6G) using the “Matchmaker” tool of ChimeraX. The alignment was used to highlight residues of the H-T interface in the AtCRY2 and their matching residues in MmCRY2.

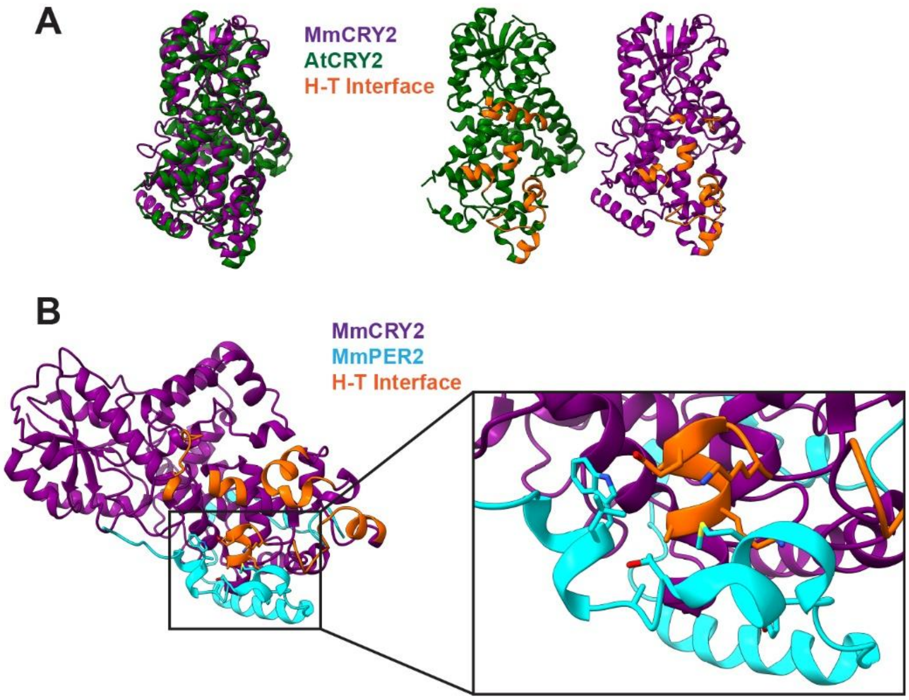

Figure S4. Speculated model for occlusion of the H-T interface by PER2 in mammalian CRY2. (A) (left) Structural alignment of AtCRY2 (PDB: 6K8I) with MmCRY2 (PDB: 4I6G) showing conserved overall fold of the PHR domains. (right) Separate renderings of MmCRY2 (purple, right) and AtCRY2 (green, left) showing the residues involved in the H-T interface in AtCRY2 (orange). (B) Crystal structure (PDB: 4U8H) of MmCRY2 (purple) in complex with the CRY-binding domain of murine PER2 (cyan), implying partial occlusion of the putative H-T interface (orange).
